# Prognosis of Cirrhotic Patients After Osteoporotic Femoral Neck Fracture

**DOI:** 10.3390/jcm13226701

**Published:** 2024-11-07

**Authors:** Aviya Muallem, Leonid Kandel, Zvi Ackerman

**Affiliations:** 1 Department of Orthopedic Surgery, Hadassah-Hebrew University Medical Center, Mount Scopus Campus and the Hebrew University-Hadassah Medical School, Jerusalem, Israel; aviya.muallem@gmail.com (A.M.); kandel@hadassah.org.il (L.K.); 2 Department of Medicine, Hadassah-Hebrew University Medical Center, Mount Scopus Campus and the Hebrew University-Hadassah Medical School, Jerusalem, Israel

**Keywords:** decompensated cirrhosis, FIB-4, osteoporosis, hip fracture, all-cause mortality

## Abstract

**Introduction and Objectives**: Osteoporotic hip fractures in cirrhotic subjects are associated with increased post-fracture mortality. Our aim was to identify unfavorable factors that were associated with increased post-fracture mortality. **Patients and Methods**: We employed a retrospective evaluation of the short- and long-term prognosis of cirrhotic patients that were admitted with a hip fracture to our institution. **Results**: A total of 77 cirrhotic and 81 control patients were included. The majority of the patients who died either during the initial three months or during one year of follow-up after the hip fracture were cirrhotic. The patients that did not survive the three-month period suffered from decompensated cirrhosis. The variables that were associated upon multivariate analysis with increased one-year all-cause mortality in both the control and cirrhotic patients were the presence of either cirrhosis, congestive heart failure or low hemoglobin levels upon admission. The variables that were associated upon univariate analysis with increased one-year all-cause mortality only in the cirrhotic patients were the patient’s age, the presence of hepatic encephalopathy, as well as the levels of serum albumin, PT (in %) and FIB-4. Our multivariate analysis disclosed that the admission level of PT (in %) was the only parameter that was associated with one-year all-cause mortality among the cirrhotic patients (adjusted OR 0.962, CI: 0.928–0.996, *p* = 0.029). **Conclusions**: Patients with decompensated cirrhosis are at an increased risk of dying during the first year after an osteoporotic hip fracture. Cirrhotic patients with osteoporosis who are at risk of hip fractures should be identified and measures to prevent this complication should be implemented.

## 1. Introduction

Osteoporotic hip fractures are an increasingly common heath problem among the general population and especially among older individuals [1,2,3]. An increase in short-term and long-term mortality after a hip fracture in the general population was widely reported. The mortality rate in the post-fracture period varies from 16% to 25% after three months and 20% to 40% after one year from the hip fracture [1,2,3,4]. Increasing age, male gender, being institutionalized, and the presence of various pre-fracture co-morbidities were reported to increase the short-term and long-term mortality in individuals from the general population that suffer from hip fractures [2,3,4,5,6]. The co-morbidities that were reported to increase mortality after a hip fracture include the following: liver disease (mild to severe), chronic renal failure, chronic obstructive pulmonary disease, dementia, diabetes mellitus, visual and hearing impairment and incontinence [2,3,4].

Liver cirrhosis is a multisystem disorder. Patients with cirrhosis suffer, in addition to liver dysfunction, from dysfunction of multiple organs like the kidneys, brain, heart, lungs, systemic circulation, intestine, immune system, adrenal glands, thyroid, reproductive system and musculoskeletal system [7]. The dysfunction of the musculoskeletal system was expressed as sarcopenia, and/or osteopenia and/or osteoporosis with an increased risk for bone fractures in multiple sites like the hip, spine and limbs [8,9].

The pathophysiology for the increased occurrence of osteoporosis and osteoporotic fractures in cirrhotic patients is complex and includes the presence of the following: hyperbilirubinemia (that was reported to cause the inhibition of osteoblast proliferation and impaired osteoblastogenesis, and increase the viability and decrease the apoptosis of osteoclasts), low bone formation rate, exposure to alcohol, malnutrition, physical inactivity, sarcopenia, chronic systemic inflammation, hepatic encephalopathy, cognitive impairment and recurrent falls [8,9,10,11,12,13,14,15,16].

Increased risk of osteoporotic fractures, at various bony locations, in patients with cirrhosis due to various etiologies like alcoholic, primary biliary cholangitis (PBC) and non-alcoholic fatty liver disease (NAFLD) was reported [8,9,17,18,19,20].

In the last decades, the prevalence of NAFLD has increased and it is expected that in the near future NAFLD will become the most common etiology for chronic liver disease and cirrhosis (Reviewed in [21,22]). Clinical studies published recently have reported a decrease in the rate of bone production and an increased rate of bone absorption in elderly NAFLD patients. Moreover, such patients have been found to have a higher 10-year probability of major osteoporotic fracture, especially in those with sarcopenia (Reviewed in [21,22]).

It has been suggested that there are potential associations between NAFLD and osteoporosis, linking the pathogenesis of NAFLD to the process of bone remodeling. The proposed pathophysiological mechanisms shared by both NAFLD and osteoporosis are as follows: Vitamin D deficiency, chronic inflammation, insulin resistance, increased gut barrier permeability due to gut microbiota dysbiosis, changes in bile acid signaling and in the content and profile of short-chain fatty acids, as well as the consumption of a Western diet and decreased physical activity. It was also suggested that bone tissue metabolites, due to their effect on lipid and glucose homeostasis, as well as bone turnover and liver fibrosis, may contribute to decreased bone mass in NAFLD (Reviewed in [22]).

In recent years an increased 30-day and one-year all-cause mortality in cirrhotic patients during the post-fracture period was reported [9,17]. This excess of observed mortality was not related to the skeletal location of the fracture or to the presumed fracture mechanism [9,17].

The aim of the current study was to evaluate retrospectively the three-month and one-year all-cause mortality of cirrhotic patients that suffered from a liver disease due to several etiologies and were admitted with an osteoporotic hip fracture to our institution. In addition, we intended to investigate the clinical and laboratory parameters that were associated with either the three-month or one-year all-cause mortality. Identifying cirrhotic patients that suffered from a hip fracture and were found to have unfavorable predictive risk factors may necessitate a change in the therapeutic approach in order to reduce the devastating effect of the hip fracture in these patients.

Moreover, identifying elderly cirrhotic patients with osteoporosis, who are at risk of recurrent falls and of a hip fracture and are found to suffer from unfavorable predictive risk factors for early mortality, should encourage their primary care physicians to implement measures to prevent hip fractures among their patients.

## 2. Patients and Methods

### 2.1. Setting and Population

This was a retrospective case control study. Data was collected from patients with hip fracture, who were admitted to one of the two campuses of the Hadassah-Hebrew University Medical Center in Jerusalem, Israel during the period from 1999 to 2018. Patients who were diagnosed pre-operatively with cirrhosis were included in the study group. The diagnosis of cirrhosis was based on the appropriate clinical, biochemical, and serological tests, in addition to various radiological imaging (including abdominal ultrasound and/or computerized tomography and/or abdominal and hepatic magnetic resonance imaging), histological findings and the presence of signs of significant portal hypertension (esophageal and/or gastric varices and/or ascites) and hepatic encephalopathy. The control group included patients with osteoporotic hip fractures without cirrhosis, matched by age, sex, procedure type and year of surgery.

Medical charts of patients from the study groups were reviewed for the following parameters: demographics (including gender and age of the patient), the date of the hip fracture and the type of surgery (if it was performed), the etiology of the cirrhosis, the presence of signs of significant portal hypertension and hepatic encephalopathy, the presence of additional background medical conditions (like diabetes mellitus, hypertension, ischemic heart disease, congestive heart failure, dementia, chronic renal failure and cerebrovascular disease), administration of specific medications (for the control of diabetes mellitus, anticoagulation, anti-platelet aggregation and medications for the control of viral hepatitis), and laboratory parameters (like liver enzymes, bilirubin, albumin, prothrombin time (PT; expressed in %), renal function and levels of hemoglobin at admission and discharge). Operative data (type of operation), administration of pre-operative or post-operative blood transfusions and survival at 3 months and 1 year after the admission for hip fracture. Since many participants of this study received their medical follow-up after their discharge from our hospital at different medical facilities, we were unable to obtain reliable information about their exact causes of death. Mortality data were obtained from the records of the Ministry of Interior. This data contains only the date of death without any additional medical information.

For each patient from the cirrhotic group, the scores that were calculated for the assessment of liver fibrosis on the Fibrosis-4 Index (FIB-4) and for the prediction of the peri-operative risk of cirrhotic patients included the following: Child–Turcotte–Pugh (CTP) score, Model for End-stage Liver Disease (MELD) and MELD-Sodium (MELD-NA) [23].

### 2.2. Statistical Analysis

In order to estimate the sample size, we used an assumption (based on previous studies) that the one-year mortality after hip fracture, in the general population, is about 20% [1,2,3,4]. Assuming that the true probability of one-year mortality in cirrhotic patients after a hip fracture is 40%, a sample size of 64 cirrhotic patients and 64 control subjects provided 80% power to reject the null hypothesis that the mortality rates are equal with a 0.05 probability of type 1 error. Comparisons between groups were performed using 2-sample *t*-tests or the Mann-Whitney nonparametric test for continuous variables and X^2^ or Fisher’s exact test for categorical variables. Variables noted to be significant upon univariate analysis were evaluated in a multivariate model using forward stepwise logistic regression analysis. Odds ratios (ORs) are reported with 95% confidence intervals. All statistical tests were two-sided, and a *p* value of <0.05 was considered statistically significant. The statistical program used for the analyses was the Statistical Product and Service Solutions (SPSS) 22.0 software.

### 2.3. Ethical Considerations

The research proposal was approved by the Helsinki Committee of the Haddasah Medical Organization (HMO), (No: 0637-19-HMO). Due to the retrospective nature of the present research, no personal informed consent was required.

## 3. Results

A total of 158 patients who were admitted to our medical centers because of an osteoporotic hip fracture were included in the study; 77 were cirrhotic and 81 were included in the control group. All of the patients that were included in cirrhotic group were diagnosed with cirrhosis a long time before their admission for the hip fracture. Most of the study participants were female (66.5%; Table 1). The etiology of the liver disease among our cirrhotic patient population was as follows: cryptogenic—24 patients (31.2%), Hepatitis C virus infection—17 patients (22.1%), non-alcoholic steatohepatitis—11 patients (14.3%), alcoholic—6 patients (7.8%), Hepatitis B virus infection—6 patients (7.8%), PBC—6 patients (7.8%), cardiac cirrhosis—4 patients (5.2%) and autoimmune liver disease—3 patients (3.9%). Documentation of hepatic encephalopathy was present in 20 patients (26%), esophageal varices in 14 patients (18.2%) and ascites in 12 patients (15.6%).

After admission, only 149 of our patient population (70 from the cirrhotic group and 79 from the control group) were found to be able to undergo an operative procedure. The operative procedures that were performed in our patient populations from both groups included the following: total hip replacements, partial hip replacements, and open and closed reductions. These procedures were performed under either general or regional anesthesia (epidural or spinal). The decisions on whether to operate or not and the type of operation and anesthesia to perform in the specific patient were made independently by the attending orthopedic surgeons that were providing patient care in the two medical centers. At that time, no research was envisioned.

The majority of the patients who died during the initial three months or during one year of follow-up after the hip fracture were cirrhotic (15/77 vs. 4/81. *p* = 0.005, 32/77 vs. 8/81. *p* < 0.001, respectively; Table 2). The difference in all-cause mortality between the cirrhotic and the control patients remained constant even after the exclusion of patients under the age of 65 and those who, for various reasons, did not undergo any operative procedure after the hip fracture (Table 2).

### 3.1. Risk Factors for Three-Month and One-Year All-Cause Mortality in the Combined Patient Population (Cirrhotic and Control)

The values of admission laboratory parameters that were observed in the cirrhotic and control patients who did not survive the follow-up periods of either three months or one year after the hip fracture were different than those observed in the patients that survived these follow-up periods. The patients who did not survive the three-month period had lower levels of albumin, sodium, hemoglobin, platelets and PT (%) and higher levels of creatinine and bilirubin (Supplementary Table S1). The patients who did not survive the one year of follow-up had lower levels of albumin, hemoglobin, platelets and PT (%), and higher levels of aspartate aminotransferase and bilirubin (Supplementary Table S2).

Our univariate analysis disclosed that the clinical variables that were associated with three-month all-cause mortality in both groups of patients were as follows: the presence of either ascites (*p* = 0.04), encephalopathy (*p* < 0.001) or esophageal varices (*p* = 0.015), in addition to the patient not being treated with anti-platelets agents (*p* = 0.05), not being able to undergo an operative procedure (*p =* 0.001), or being able to tolerate only regional anesthesia during the hip surgery (*p =* 0.023), (Table 3).

The clinical variables that were found to be associated, by univariate analysis, with one-year all-cause mortality in both groups of patients included the following: presence of either congestive heart failure (*p* = 0.029), ascites (*p* = 0.012), encephalopathy (*p* < 0.001) or esophageal varices (*p* = 0.047), and the finding that a patient was not able to undergo an operative procedure (*p* = 0.046) (Table 3).

The parameters that were found upon univariate analysis to be associated with one-year all-cause mortality in both groups of patients were entered into a multivariate analysis. Since none of the control patients suffered from cirrhosis and its consequences, the parameters that were directly related to the presence of advanced and decompensated liver disease like ascites, encephalopathy, and esophageal varices, as well as the levels of albumin, bilirubin and PT (%), were not introduced into this multivariate analysis. The only variables that were found to be significantly associated with one-year all-cause mortality were the presence of cirrhosis, congestive heart failure, and admission hemoglobin levels (Table 4).

### 3.2. Risk Factors for One-Year All-Cause Mortality in the Cirrhotic Patients

In order to study the variables that were pertinent for the prediction of one-year all- cause mortality, a univariate analysis of clinical variables was calculated only in the cirrhotic patients. In this analysis, the presence of hepatic encephalopathy was the only variable that was found to be associated with one-year all-cause mortality (*p* = 0.013), (Table 5). Additional continuous variables that were found upon univariate analysis to be statistically significant in the prediction of one-year all-cause only in the cirrhotic patients were the patient’s age (*p* = 0.022) and the levels of the following parameters measured at admission: serum albumin (*p* = 0.016), PT (%; *p* = 0.034) and the calculated values of FIB-4 *(p* = 0.05), (Supplementary Table S3).

The above variables, which were found to be predictive of one-year all-cause mortality, were introduced into a model of multivariable regression analysis. The only parameter that was found to be associated with one-year all-cause mortality was the level of PT (%). As the value of PT increased, the probability for one-year all-cause mortality decreased (adjusted OR 0.962, CI: 0.928–0.996, *p* = 0.029).

## 4. Discussion

### 4.1. Risk Factors for Three-Month and One-Year All-Cause Mortality in the Combined Patient Population (Cirrhotic and Control)

In the present retrospective cohort study that involved 158 patients that were admitted to our medical centers in Jerusalem because of an osteoporotic hip fracture, we were able to observe that, in comparison to control patients, cirrhotic patients suffered from higher all-cause mortality rates after three months and after one year of follow-up (19.5% vs. 4.9% and 41.6% vs. 9.9%, respectively). The three-month and one-year all-cause mortality rates that were observed in our control patients were lower than those reported previously [1,2,3,4], but similar to recently reported studies [24,25]. It had been suggested that the recently observed decline in mortality rates in the follow-up period after a hip fracture may be related to improvements in both pre- and post-operative medical treatment and in rehabilitation programs of patients with hip fractures [26].

In recent years, several studies reported the short- and long-term prognosis after osteoporotic hip fractures in cirrhotic patients [5,6,10,18,19,20,24,25]. Tseng and colleagues from Taiwan reported that geriatric patients with cirrhosis that underwent a hip fracture surgical repair had a high risk for in-hospital mortality [10]. Otete and colleagues reported that 30-day mortality after a hip fracture among English and Danish alcoholic cirrhotic and control patients ranged from 11.1% to 10% and 5.0% to 6.6%, respectively [19]. In another study from Taiwan, Chang and colleagues reported that the three-month and one-year all-cause mortality rates after a hip fracture in cirrhotic patients compared to control patients were 2.3 and 2.6-fold higher, respectively. The etiology of cirrhosis was not recorded in this study; however, it was assumed that in most of the cirrhotic patients the etiology of the liver disease was viral hepatitis [20]. Montomoli and colleagues reported that the 30-day all-cause mortality rate after a hip fracture in Danish cirrhotic patients and in patients without liver disease was 12.6% and 9.7%, respectively. Among the patients who survived the first 30 days, the mortality rate during the 31–365 days of follow-up was 26.4% among the cirrhotic patients and 19.4% among the control patients [24]. In all of the above reported studies, no correlation between the short- and long-term prognosis and the severity of the liver disease was reported. Hundersmarck and colleges from the USA reported their experience with 128 cirrhotic patients who were admitted because of hip fractures. The post-fracture prognosis was associated with the degree of liver dysfunction; patients with decompensated cirrhosis that was characterized by the presence of either ascites and/or encephalopathy and/or a history of variceal bleeding had a three-month and one-year mortality rate of 24% and 53%, respectively. Patients with compensated liver disease had a three-month and one-year mortality rate of 8% and 15%, respectively [25]. Similar findings were reported in our study. The majority of patients with a hip fracture who died during either the first three months or during the follow-up period of one year were cirrhotic. Moreover, those patients who did not survive either the initial three months or the one-year follow-up period had clinical and laboratory characteristics of advanced and decompensated cirrhosis. Our multivariate analysis of the clinical and laboratory variables that were associated with all-cause mortality one year after a hip fracture in both cirrhotic and control patients disclosed three variables that were found to be significantly associated with one-year all-cause mortality: the presence of cirrhosis, congestive heart failure, and admission hemoglobin levels.

Hepatic decompensation with an increase in morbidity and mortality after various surgical procedures is a commonly reported event in patients with previously known or unknown cirrhosis [27,28]. The mechanisms that are responsible for the precipitation of hepatic decompensation and the increase in post-operative morbidity and mortality in cirrhotic patients are unknown. It can be hypothesized that surgical complications such as infection and bleeding, which are more common and more severe in patients with advanced cirrhosis, may precipitate the dysfunction of multiple organs and an increase in the incidence of additional events such as septic shock, various hematological complications (like thrombotic processes, increased fibrinolysis or defects in platelets function), an increase in portal hypertension (with formation of ascites, hepatic encephalopathy and acute kidney injury) and various cardiopulmonary complications (reviewed in [29]).

Similar to our findings, Cha and colleagues have reported that elderly patients with hip fractures and congestive heart failure are at risk for early peri-operative mortality, Patients with a hip fracture and congestive heart failure had an increased one-year cumulative mortality compared to subjects without congestive heart failure (25.2% vs. 12.9%, respectively) [30].

An additional risk factor that was associated with increased one-year all-cause mortality in the combined group of patients (cirrhotic and control patients) was their admission hemoglobin levels. Pre-operative anemia in elderly patients with hip fractures can be the end result of several pathologies including acute blood loss due to the bone and soft tissue damage, along with the underlying chronic co-morbidities and malnutrition that are often present in patients with hip fractures. It was reported that pre-operative anemia (even mild anemia) was associated with major post-operative complications and death in hip fracture patients even up to six months after surgery [31,32]. The pre-operative correction of admission hemoglobin levels to >10 g/dL by blood transfusion was found to be associated with a 50% reduction in one-year all-cause mortality in osteoporotic hip fracture patients [33].

### 4.2. Risk Factors for One-Year All-Cause Mortality in the Cirrhotic Patients

Further analyses that were performed only among the cirrhotic patients identified several clinical and laboratory parameters, which were found to be associated upon univariate analysis with one-year all-cause mortality. These included the age of the patients, the presence of hepatic encephalopathy and the levels upon admission of the following parameters: serum albumin, PT (%), and the calculated value of FIB-4.

The age of cirrhotic patients, serum albumin and prothrombin time are frequently reported as predictors of prognosis in patients with compensated and decompensated cirrhosis [34]. Older patients with cirrhosis have a reduced life expectancy. This may be related to the presence of multiple co-morbidities with the increasing age of the cirrhotic patient. Moreover, sarcopenia and osteoporosis are more frequently present in older cirrhotic patients and may contribute to their frailty [6,35].

The presence of hepatic encephalopathy occurs relatively late in the course of cirrhosis. In order to present with hepatic encephalopathy, the cirrhotic patient needs to have a substantial decrease in hepatic function and the presence of significant porto-systemic shunting, which allows various substances (including ammonia) to get into the systemic circulation and later into the cerebral circulation [36]. Stewart and colleagues demonstrated that although the stage of hepatic encephalopathy correlates with other markers of cirrhosis severity like MELD, hepatic encephalopathy affects patient survival independent of MELD [36]. Moreover, Tapper and colleagues reported that age may be a major determinant of patients’ survival after the diagnosis of hepatic encephalopathy. Patients with cirrhosis aged ≥65 had an overall median survival of 0.95 years, compared to 2.5 years for those <65 years old [37]. It should be noted that the presence of hepatic encephalopathy even at minimal stages may increase the risk of falls [38].

Serum albumin levels are considered a “liver function test”. Moreover, serum albumin levels are an integral component of the CTP score that assesses the prognosis of patients with cirrhosis [23]. Albumin is synthesized in the liver; however, serum albumin levels depend not only on the synthetic liver function but on additional factors like the nutritional status, presence of sepsis, systemic inflammatory disorders, and urinary and gastrointestinal losses. Similar to our findings, Mahmud and colleagues recently reported that pre-operative albumin levels were an important component in predicting post-operative prognoses [39] Nyberg and colleagues studied 853 cirrhotic patients that underwent orthopedic surgeries. A decrease in serum albumin levels was identified as being associated with hepatic decompensation at 90 days post-surgery [40].

In our study, the calculated FIB-4 at presentation was a predictive tool and correlated with the one-year survival of the cirrhotic patients after a hip fracture. Similar results were reported by others. Zelber-Sagi and colleagues reported their observations in a historical cohort of 19,861 subjects who underwent general anesthesia for various surgical procedures and were not known to have an overt liver disease. Calculated high FIB-4 values (>2.67), which served as a marker of advanced liver fibrosis, was associated with increased mortality during either the operative procedure or the hospitalization, or within 30 days of the surgical procedure [41].

The above variables, which were found to be predictive of one-year all-cause mortality, were introduced into a model of multivariable regression analysis. The only parameter that was found to be associated with one-year all-cause mortality was the level of PT (%). As the value of PT (%) increased, the probability for one-year all-cause mortality decreased (adjusted OR 0.962, CI: 0.928–0.996, *p* = 0.029). The important role of PT (%) in the prediction of post-operative mortality was also described by others. A deranged level of pre-operative PT (%) was one of the parameters that predicted post-operative mortality among cirrhotic patients that underwent a variety of operative procedures [42,43]. PT is recognized to be a reliable marker of protein synthetic function of the liver, like some of the clotting factors, which have a short half-life within the circulation [44]. Deficiency of these factors due to poor synthetic activity reflects disease progression in patients with cirrhosis, and hence it is an important parameter in various reliable prognostic models of cirrhosis like the CTP and MELD scores. These scores have an important role in the accurate prediction of the severity of liver disease and effectively assess the risk of mortality [23,45,46].

In our study, the pre-operative values of CTP and MELD scores were not different between the cirrhotic patients that survived and those that did not survive the one-year follow-up period after the hip surgery. Our inability to use the CTP and MELD scores for the prediction of long-term prognosis among the cirrhotic patients may be related to either the relatively low sample size of our cirrhotic patients and/or to the lack of uniformity among our patients regarding the following: (a) the etiology of liver disease, its severity and its complications. (b) the presence of additional medical co-morbidities, and (c) the type of hip surgery that our patients underwent.

Our article has several limitations. Firstly, this is a retrospective observational study and as such is subject to selection bias. Moreover, due to the nature of the study, it is possible that some patients with undiagnosed compensated cirrhosis that had an uneventful post-operative course were not identified and were not assigned to the cirrhotic group [41]. Secondly, this study was a single-centered study with a relatively small cohort of cirrhotic patients due to seven different etiologies. It is known that the risk for developing cirrhosis with progressive liver failure and severe portal hypertension, the risk for developing additional co-morbidities, the risk for osteoporotic femoral neck fracture, the risk for morbidity from the fracture and the risk for mortality from complications that accompany the femoral neck fracture in patients with PBC may be totally different from those that are found in patients with liver diseases due to a different etiology [17,47,48]. Therefore, the results of our study might not be generalized to other populations with a different mix of etiologies for their liver disease. Thirdly, this study design does not allow us to establish the etiologies of the increased three-month and one-year all-cause mortality in our cirrhotic patient population. Despite these limitations, our study has some strengths. All the laboratory testing during the study period was performed by the same laboratory, using the same laboratory kits and techniques, and the data presented in this manuscript were retrieved from the archives of our institution and not from multiple sources.

## 5. Conclusions

Our study showed that patients with decompensated cirrhosis, irrespective of its etiology, are at an increased risk of dying during the first year after an osteoporotic hip fracture. Orthopedic surgeons should make an effort to identify patients with advanced liver disease among the patients that are admitted to their care because of hip fractures. The implementation of measures to reduce the risk of these patients for dying in both the early and late post-operative period should be carried out.

Moreover, the results of our study should also encourage primary care physicians and geriatricians to identify elderly cirrhotic patients with osteoporosis who are at risk of recurrent falls and of a hip fracture and to implement measures to prevent these complications.

## Figures and Tables

**Table 1:** Gender and operative status among different study groups.

Characteristics		Cirrhotic Group (*n* = 77)	Control Group (*n* = 81)	Combined (*n* = 158)
Gender, *n* (%)	Female	52 (67.5)	53 (65.4)	105 (66.5)
	Male	25 (32.5)	28 (34.6)	53 (33.5)
Surgery, *n* (%)	Yes	70 (91)	79 (97.5)	149 (94.3)
	No	7 (9)	2 (2.5)	9 (5.7)

**Table 2:** All-cause mortality rates among combined patient population (cirrhotic and control).

Characteristics		Cirrhotic Group (*n* = 77)	Control Group (*n* = 81)	*p*-Value
All patients	Mortality at 3 months, *n* (%)	15/77 (19.5)	4/81 (4.9)	0.005
	Mortality at 1 year, *n* (%)	32/77 (41.6)	8/81 (9.9)	<0.001
Age > 65 years	Mortality at 3 months, *n* (%)	12/62 (19.4)	4/72 (5.6)	0.014
	Mortality at 1 year, *n* (%)	28/62 (45.2)	7/72 (9.7)	<0.001
Patient underwent an operative procedure	Mortality at 3 months, *n* (%)	10/70 (14.3)	4/79 (5.1)	0.054
	Mortality at 1 year, *n* (%)	27/70 (38.6)	8/79 (10.1)	<0.001

**Table 3:** Univariate association between clinical variables and all-cause mortality at three months and one year after hip fracture in combined patient population (cirrhotic and control).

Variable	*p*-Value for Mortality at 3 Months	*p*-Value for Mortality at 1 Year
Male	0.174	0.871
Diabetes mellitus	0.051	0.066
Hypertension	0.914	0.76
Ischemic heart disease	1.0	0.633
Congestive heart failure	0.318	0.029
Dementia	0.247	0.113
Chronic renal failure	0.062	0.343
S/P cerebrovascular accident	1.0	0.972
Ascites	0.04	0.012
Hepatic encephalopathy	0.001>	0.001>
Esophageal varices	0.015	0.047
Blood transfusion before surgery	0.626	0.496
Blood transfusion post-surgery	0.708	0.125
*Rx* with anti-coagulation	1.0	0.762
*Rx* without anti-platelet	0.05	0.091
Patient not being able to undergo hip surgery	0.001	0.046
Type of surgery	0.318	0.396
Having regional anesthesia	0.023	0.092

**Table 4:** Multivariate analysis of clinical and laboratory variables associated with all-cause mortality one year after hip fracture in both cirrhotic and control patients.

Characteristics	Adjusted OR	95% CI	*p*-Value
Cirrhosis	4.646	1.854–11.641	0.001
Congestive heart failure	2.887	1.001–8.334	0.050
Hemoglobin at admission	1.304	0.606–0.969	0.026

**Table 5:** Univariate association between clinical variables and one-year all-cause mortality after hip fracture in cirrhotic patients.

Variable	*p*-Value
Male	0.847
Diabetes mellitus	0.205
Hypertension	0.375
Ischemic heart disease	0.554
Congestive heart failure	0.191
Chronic renal failure	0.924
S/P cerebrovascular accident	0.728
Ascites	0.22
Hepatic encephalopathy	0.013
Esophageal varices	0.479
Blood transfusion pre-surgery	1.0
Blood transfusion post-surgery	0.129
*Rx* with anti-coagulation	1.0
*Rx* with anti-platelet	0.631
Patient underwent hip surgery	0.12
Type of surgery	0.858
Type of anesthesia	0.233

## Data Availability

The data that support the findings of this study are available from the corresponding author upon reasonable request.

## Supplementary Materials

The following supporting information can be downloaded at: https://www.mdpi.com/article/10.3390/jcm13226701/s1, Table S1: Comparison of admission clinical and laboratory parameters between alive and deceased cirrhotic and control patients three months after hip fracture; Table S2: Comparison of admission clinical and laboratory parameters between alive and deceased cirrhotic and control patients one year after hip fracture; Table S3: Comparison of admission clinical and laboratory parameters between the alive and deceased cirrhotic patients, one year after hip fracture.

## Abbreviations

PT, prothrombin time; CTP score, Child-Turcotte-Pugh score; MELD, Model for End-stage Liver Disease; MELD-NA, MELD-Sodium; ORs, Odds ratios; SPSS, Statistical Product and Service Solutions; HMO, Haddasah Medical Organization; FIB-4, Fibrosis-4 Index; CI, confidence interval; PBC, primary biliary cholangitis; NAFLD, non-alcoholic fatty liver disease.
