# Supplementary material for: Prognosis of Cirrhotic Patients After Osteoporotic Femoral Neck Fracture
Source: J Clin Med. 2024 Nov 7;13(22):6701. doi: 10.3390/jcm13226701 (PMC11595005; doi:10.3390/jcm13226701)
Supplement: Supplementary file 1 [file jcm-13-06701-s001.zip › jcm-3261494-supplementary.pdf]

Supplementary Tables

Supplementary Table S1: Comparison of admission clinical and laboratory parameters between alive and deceased cirrhotic and control patients three months after hip fracture.

Characteristics	Alive at 3 months (n=139)	Deceased at 3 months (n=19)	*P*-value
**Age (Years)**	76.3 (9.9)	76.4 (10.1)	0.870
**AST (U/L)**	50 (60)	40 (12)	0.589
**ALT (U/L)**	28 (33)	23 (12)	0.836
**Albumin (G/L)**	35 (6.0)	24 (7.0)	**<0.001**
**Creatinine (Micromole/L)**	132 (138)	144 (74)	**0.054**
**Sodium (Mmol/L)**	137 (4.0)	135 (4.0)	**0.038**
**Bilirubin (Micromole/L)**	15 (13)	25 (18)	**0.023**
**Hemoglobin (G/dL)**	11.9 (1.8)	10.5 (1.7)	**0.005**
**Platelets (10^9/L)**	197 (101)	138 (88)	**0.007**
**PT (%)**	79 (18)	56 (23)	**<0.001**

Results are expressed as mean (± SD). AST; aspartate aminotransferase. ALT; alanine aminotransferase. PT; prothrombin time.

Supplementary Table S2: Comparison of admission clinical and laboratory parameters between alive and deceased cirrhotic and control patients one year after hip fracture.

Characteristics	Alive at 1 year (n=118)	Deceased at 1 year (n=40)	P-value
**Age (Years)**	76.0 (10.3)	77.3 (8.7)	0.463
**AST (U/L)**	37 (21)	92 (105)	**0.016**
**ALT (U/L)**	23 (14)	37 (52)	0.796
**Albumin (G/L)**	36 (6.0)	29 (8.0)	**<0.001**
**Creatinine (Micromole/L)**	134 (141)	133 (104)	0.156
**Sodium (Mmol/L)**	137 (4.0)	136 (4.0)	0.229
**Bilirubin (Micromole/L)**	14 (13)	21 (15)	**0.007**
**Hemoglobin (G/dL)**	12.0 (1.7)	11.0 (2.0)	**0.003**
**Platelets (10^9/L)**	202 (103)	158 (91)	**0.008**
**PT (%)**	(18)81	65 (21)	**<0.001**

Results are expressed as mean (± SD). AST; aspartate aminotransferase. ALT; alanine aminotransferase. PT; prothrombin time.

Supplementary Table S3: Comparison of admission clinical and laboratory parameters between the alive and deceased cirrhotic patients, one year after hip fracture.

Characteristics	Alive at 1 year (n=45)	Deceased at 1 year (n=32)	*P*-value
**Age (Years)**	72.2 (9.5)	76.5 (8.5)	**0.022**
**AST (U/L)**	42 (23)	98 (108)	0.084
**ALT (U/L)**	27 (16)	38 (53)	0.522
**Albumin (G/L)**	34 (5.0)	29 (8.0)	**0.016**
**Creatinine (Micromole/L)**	150 (130)	114 (59)	0.582
**Sodium (Mmol/L)**	136 (5.0)	136 (4.0)	0.527
**Bilirubin (Micromole/L)**	17 (15)	23 (16)	0.129
**Hemoglobin (G/dL)**	11.5 (1.8)	11 (1.9)	0.089
**Platelets (10^9 /L)**	146 (72)	148 (85)	0.810
**PT (%)**	71 (18)	)20 (62	**0.034**
**MELD Score**	)5.0 (13	)4.0 (14	0.541
**MELD-Na Score**	)5.0(16	)4.0 (17	0.460
**Child-Turcotte-Pugh Score**	)1.0 (6	7 (2.0)	0.059
**FIB-4 Score**	8.6 (17.2)	11.1 (9.0)	**0.050**

Results are expressed as mean (± SD). AST; aspartate aminotransferase. ALT; alanine aminotransferase. PT; prothrombin time. MELD; model for end-stage liver disease. MELD-Na Score; MELD-Sodium
